# Activation of PI3K/Akt/mTOR signaling in the tumor stroma drives endocrine therapy-dependent breast tumor regression

**DOI:** 10.18632/oncotarget.4203

**Published:** 2015-06-08

**Authors:** María Laura Polo, Marina Riggio, María May, María Jimena Rodríguez, María Cecilia Perrone, Melody Stallings-Mann, Diego Kaen, Marlene Frost, Matthew Goetz, Judy Boughey, Claudia Lanari, Derek Radisky, Virginia Novaro

**Affiliations:** ^1^ Instituto de Biología y Medicina Experimental, Protein Kinases and Cancer Laboratory, Buenos Aires, Argentina; ^2^ Mayo Clinic Comprehensive Cancer Center, Department of Cancer Biology, Jacksonville, FL, USA; ^3^ Centro Oncológico Riojano Integral, Medical Oncology, La Rioja, Argentina; ^4^ Department of Medical Oncology, Mayo Clinic, Rochester, MN, USA; ^5^ Department of Surgery, Mayo Clinic, Rochester, MN, USA

**Keywords:** tumor stroma, neoadjuvant endocrine therapy, PI3K/Akt pathway, breast cancer

## Abstract

Improved efficacy of neoadjuvant endocrine-targeting therapies in luminal breast carcinomas could be achieved with optimal use of pathway targeting agents. In a mouse model of ductal breast carcinoma we identify a tumor regressive stromal reaction that is induced by neoadjuvant endocrine therapy. This reparative reaction is characterized by tumor neovascularization accompanied by infiltration of immune cells and carcinoma-associated fibroblasts that stain for phosphorylated ribosomal protein S6 (pS6), downstream the PI3K/Akt/mTOR pathway. While tumor variants with higher PI3K/Akt/mTOR activity respond well to a combination of endocrine and PI3K/Akt/mTOR inhibitors, tumor variants with lower PI3K/Akt/mTOR activity respond more poorly to the combination therapy than to the endocrine therapy alone, associated with inhibition of stromal pS6 and the reparative reaction. In human breast cancer xenografts we confirm that such differential sensitivity to therapy is primarily determined by the level of PI3K/Akt/mTOR in tumor cells. We further show that the clinical response of breast cancer patients undergoing neoadjuvant endocrine therapy is associated with the reparative stromal reaction. We conclude that tumor level and localization of pS6 are associated with therapeutic response in breast cancer and represent biomarkers to distinguish which tumors will benefit from the incorporation of PI3K/Akt/mTOR inhibitors with neoadjuvant endocrine therapy.

## INTRODUCTION

More than 70% of breast carcinomas are estrogen receptor (ER) and progesterone receptor (PR) positive and are thus potentially susceptible to anti-endocrine therapies. Neoadjuvant endocrine therapy is increasingly used in patients that share clinicopathological characteristics such as: high ER and PR, low Ki67, HER-2 negative, large tumor size, advanced age [1, 2] or presence of comorbidities that preclude surgery. While estrogen signaling is a key feature of breast cancer etiology, experimental and epidemiological evidence has also linked progesterone to breast cancer pathogenesis [3, 4]. *A*ntiprogestins are being tested in clinical trials as second or third line anti-endocrine therapies in advanced or metastatic breast cancer settings, reviewed in [5]. Among antiprogestins, mifepristone (MFP) has shown considerable potential. MFP binds to PR, allows dimerization of the receptor and subsequent binding to DNA [6], but prevents recruitment of transcriptional coactivators to target genes [7].

More than 50% of ER/PR-positive breast cancers contain alterations in at least one component of the PI3K/Akt/mTOR pathway, and these alterations drive cancer growth and also facilitate development of resistance to hormone prevention therapies [8, 9]. For these reasons, therapeutic agents that target the PI3K/Akt/mTOR pathway have been actively pursued, with several agents being tested in phase I to phase III studies in combination with endocrine therapy [10–12]. Based on the BOLERO-2 trial in which the addition of everolimus (RAD001), an mTOR inhibitor, improved progression free survival in aromatase inhibitor resistant breast cancer, the US FDA approved the use of everolimus in combination with exemestane in patients with metastastic ER-positive breast cancer refractory to aromatase inhibitors [13–17].

The tumor microenvironment comprises a heterogeneous population of cells including fibroblasts, adipocytes, endothelial and immune cells, and the extracellular matrix which serves as a scaffold and which provides contextual information for the above-mentioned cells [18]. Among these stromal cell types, cancer-associated fibroblasts (CAFs) have been identified as important promoters of tumor growth and progression [19], as players in development of hormone independence [20], and as potential therapeutic targets in highly desmoplastic human cancers [21]. A number of studies have identified specific elements of the tumor microenvironment that contribute to tumor proliferation, neovascularization, invasion, and metastasis [22, 23]. Moreover, differential gene expression from breast tumor stroma can be used to identify gene profiles associated with breast cancer outcomes [24]. However, the role of the tumor microenvironment in therapeutic regression remains unknown.

We have used medroxyprogesterone acetate (MPA)-induced mouse models of breast cancer [25] to define the cellular and molecular mechanisms associated with tumor regression following endocrine therapy that have allowed us to identify key microenvironmental factors that can modulate hormone dependency [26], response to endocrine therapy, and to PI3K kinase inhibitors [27]. Here, we show that endocrine therapy-induced tumor regression in hormone-dependent mammary tumors requires a stromal response that is dependent upon PI3K/Akt signaling. Our findings have significance for effective therapeutic targeting of this common breast cancer subtype.

## RESULTS

### Inhibition of PI3K/Akt enhances endocrine therapy-induced tumor regression in hormone- independent mammary carcinomas

**Figure 1:**
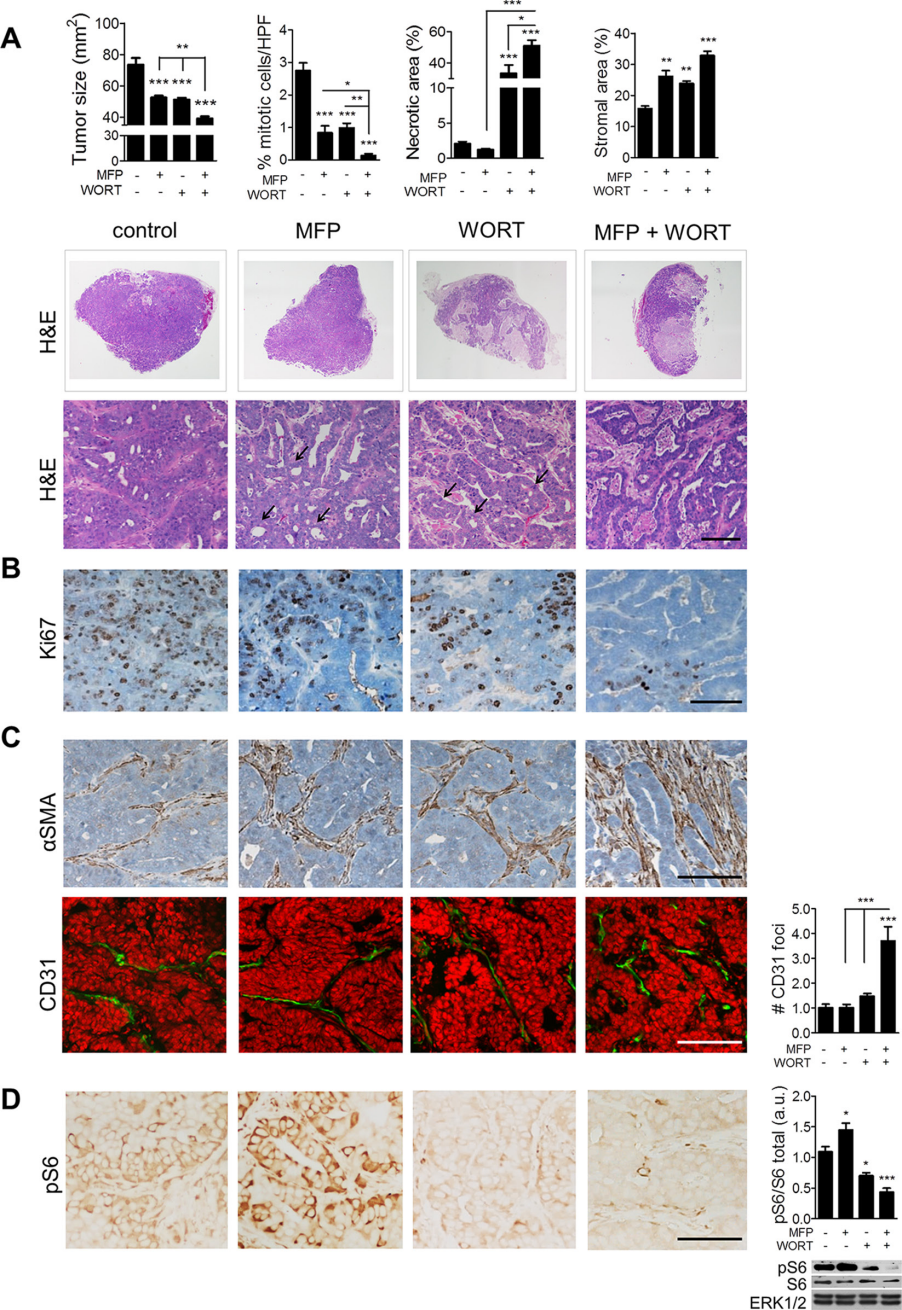
Combination of MFP and WORT improves C4-HI tumor regression C4-HI tumors of 50 mm^2^ were treated with MFP (12 mg/kg/day), WORT (1 mg/kg/day), MFP+WORT, or saline (control) for 48 h. **A. Top**: Tumor size diminished further by MFP+WORT than by MFP or WORT itself. Mitotic cells, necrotic and stromal areas were quantified in H&E. **Middle**: H&E staining in whole FFPE tumor sections reveals greater necrosis (pink areas) after combination therapy. **Bottom**: High magnification images show tissue remodeling with signs of differentiation (arrows show ductal-like structures). **B.** IHC for Ki67 confirms the quantification of mitotic cells. **C.** IHC for αSMA and IF for CD31. Nuclei were stained in red with PI. Quantification shows that the number of CD31-positive foci increased after combination therapy. **D. Left**: IHC for pS6 in Ser240/244 shows that both tumor and stromal levels diminished with WORT. Nuclei were not counterstained with hematoxilyn to evidence pS6 signal. **Right**: WB revealing total and pS6, and ERK1/2 as a loading control. Quantification shows that MFP increased whereas WORT reduced pS6 levels. Bar 100 μm.

The MPA-induced mouse model of ductal ER/PR-positive breast carcinoma generates hormone-dependent tumors that require MPA for growth (C4-HD); over time, these tumors give rise to variants that can proliferate without exogenous hormone supply and are thus designated as hormone-independent (C4-HI) [25]. Both tumor types are inhibited by the antiprogestin MFP, but differ in their activation of PI3K/Akt pathway: C4-HI tumors display higher levels of PI3K/Akt, lower PTEN, and higher response to the PI3K inhibitor LY294002, or the mTOR inhibitor rapamycin, than to endocrine inhibitors, while C4-HD tumors are more responsive to endocrine inhibitors [27]. Overactivation of PI3K/Akt pathway by myristoylated Akt1 in estrogen- or MPA-dependent tumor cells induced ER and PR activation and ligand-independent tumor growth affecting endocrine therapy sensitivity [28]. Here we investigated the role of the stroma in response to PI3K inhibition using this tumor model. We found that 48 h treatment of C4-HI tumors with the PI3K inhibitor wortmannin (WORT) significantly enhanced the response to MFP, showing decreased tumor size and cell proliferation, and increased necrosis, accompanied by increased tumor stroma and tissue differentiation (Figure 1A, 1B). The enhanced tumor regression was associated with an increase in α-smooth-muscle actin (αSMA)-positive CAFs and in CD31-positive endothelial cells (Figure 1C). Moreover, while MFP treatment alone increased levels of pS6, these were effectively inhibited in mice treated with WORT (Figure 1D).

The additive effect of WORT and MFP was also found on isolated C4-HI tumor cells growing on 3D Matrigel (Supplementary Figure S1A). As with the mouse transplantation experiments, the combination of MFP+WORT had greater proapoptotic effect than either MFP or WORT alone, as observed by phase microscopy, and by acridine orange and ethidium bromide (AO/BE) (Supplementary Figure S1B) and caspase 3 staining (Supplementary Figure S1C). These results confirm that in C4-HI tumors, the additive proapoptotic effect of WORT and MFP that contribute to regression affects directly tumor cells.

### Stromal activation of PI3K/Akt is associated with tissue remodeling processes that drive therapeutic response in hormone-dependent mammary tumors

**Figure 2:**
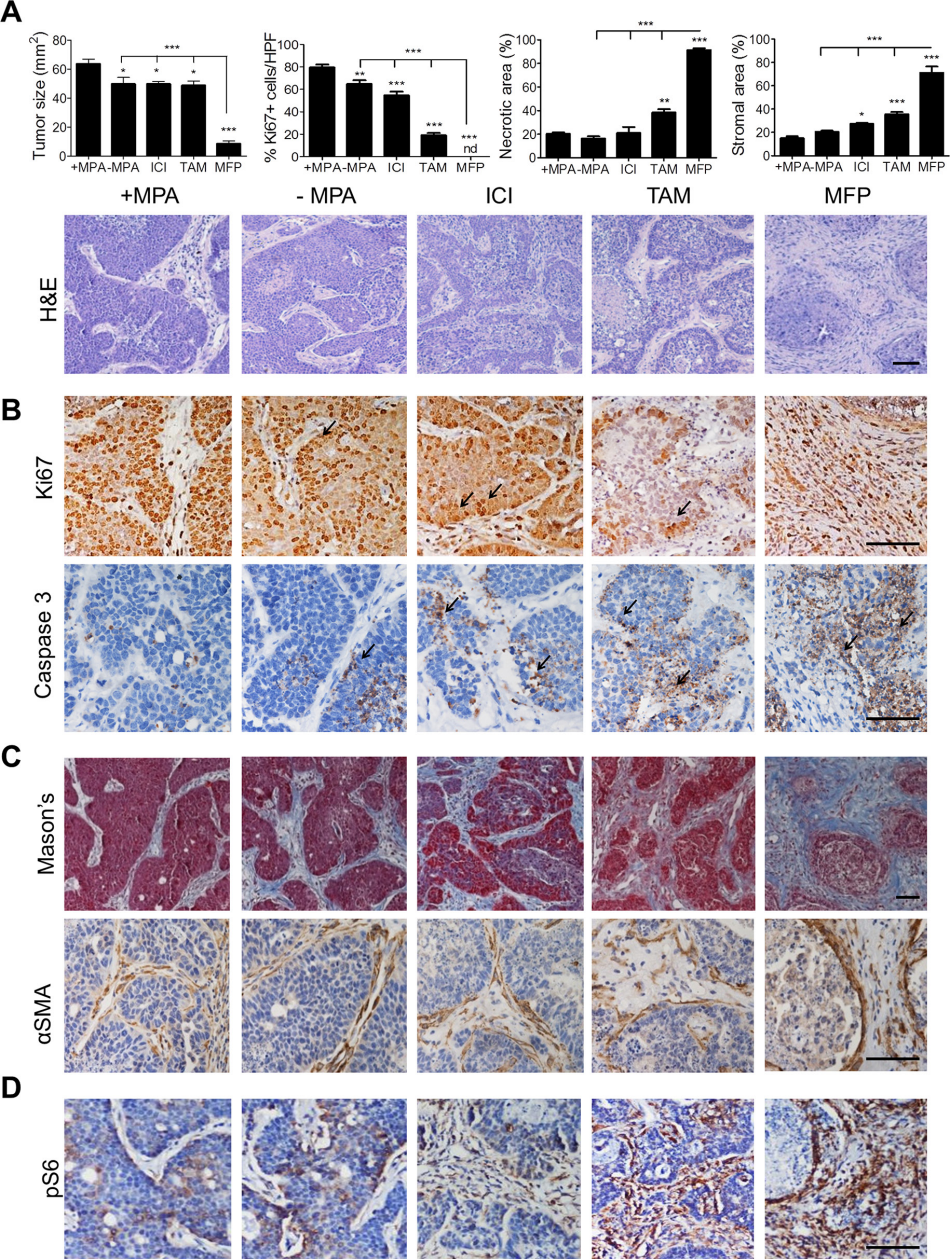
MFP is the endocrine agent that induces the strongest stromal reaction and C4-HD tumor regression When C4-HD tumors reached 50 mm^2^, MPA depot was removed and mice were treated with ICI (5 mg), TAM (5mg/kg/day), MFP or saline (−MPA) for 48 h. +MPA group was sham-operated and treated with saline. **A. Top**: Final tumor size, % Ki67-positive cells, necrotic and stromal areas were quantified in IHC or H&E. **Bottom**: H&E staining reveals that C4-HD endocrine-treated tumor displayed tissue remodeling with intense stromal invasion. **B.** IHC for Ki67 and caspase 3. Arrows indicate that in residual tumor tissue both proliferative (Ki67-positive) and apoptotic (caspase 3-positive) cells were localized to the epithelial edge in contact with the invading stroma. **C. Top**: Mason's Trichrome (blue: collagen-rich fibrillary stroma; red: parenchymal nuclei) confirmed the composition of regressive stroma. **Bottom**: IHC for αSMA shows intense signal in the regressive stromal edge facing residual tumor. **D.** pS6 staining after treatment decreased in C4-HD tumor parenchyma and increased in tumor stroma. Bar 100 μm.

We next evaluated αSMA- and CD31-positive stromal cells as biomarkers of tumor regression in hormone-dependent tumors. C4-HD tumors require MPA to grow [25] and regressed when the MPA pellet was removed or after 48 h exposure to antiestrogens such as tamoxifen citrate (TAM) or Fulvestrant (ICI182, 780; ICI), although treatment with the antiprogestin MFP induced larger tumor shrinkage (Figure 2A). While the tumor parenchyma underwent cytostasis, apoptosis and necrosis, the stromal tissue showed signs of expansion and activation, and the stromal area reflected the effectiveness of the antitumor treatments (Figure 2A–2C). We detected proliferative and apoptotic cells next to the regressive stroma, suggesting that different populations of tumor cells coexist within the tumor parenchyma in contact with the regressive stroma. With more effective therapeutic response, activation of Akt (not shown) and S6 (Figure 2D) became almost undetectable in residual tumor parenchyma but was substantially increased in the tumor stroma.

**Figure 3:**
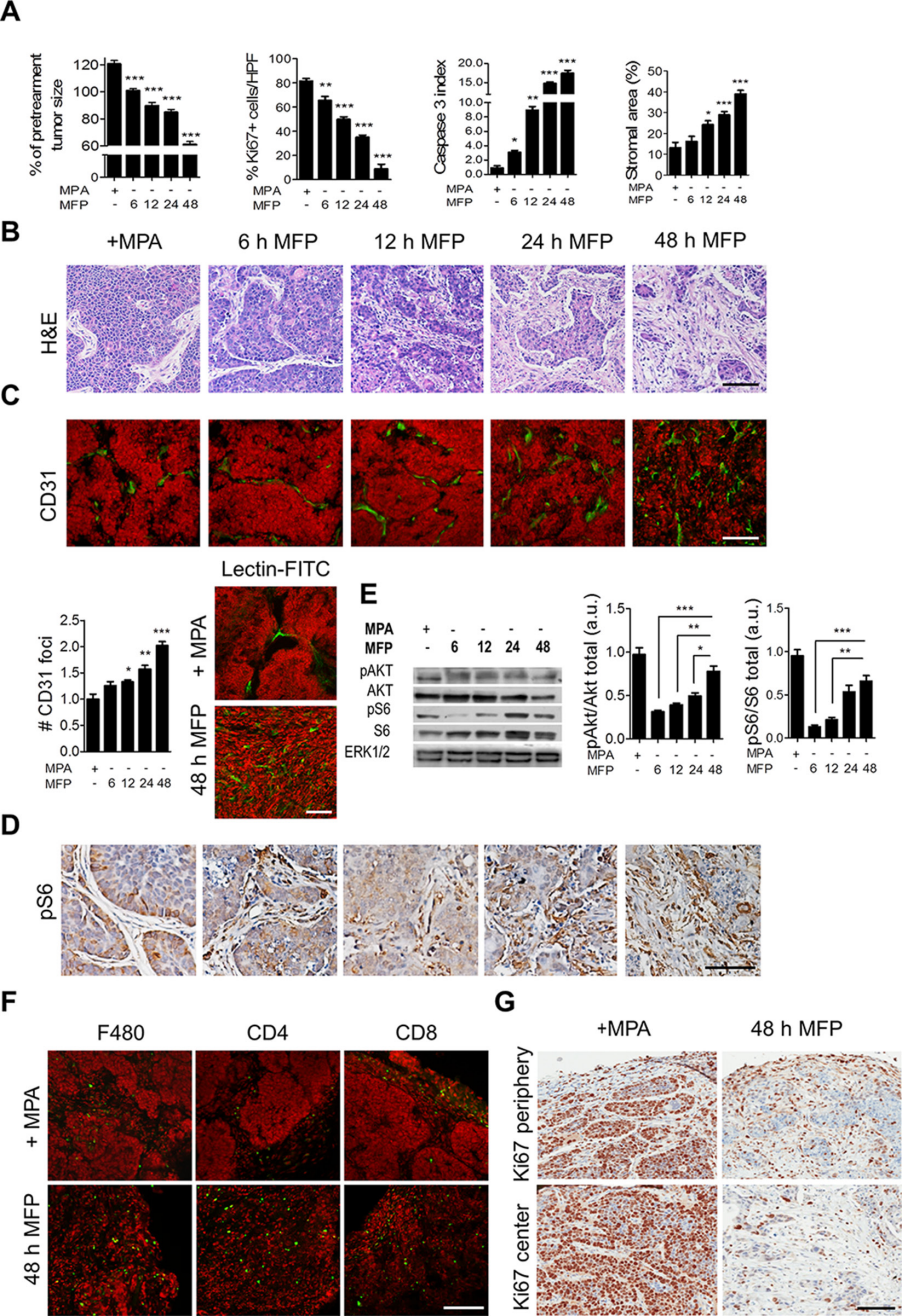
Time course and cellular mechanisms involved in stromal reaction in C4-HD tumors C4-HD tumors were treated with MFP for 6, 12, 24 or 48 h. **A.** Quantification of tumor size (% of pretreatment size), % Ki67-positive cells, caspase 3 and stromal indexes. Tumor's shrinkage, cytostasis, apoptosis and stromal area increased after MFP treatment in a time-dependent fashion. **B.** H&E staining shows tissue remodeling. **C. Top**: IF for CD31 with nuclei stained in red. **Bottom, left**: quantification of CD31 foci. **Bottom, right**: labeling with fluorescent lectins. **D.** IHC shows pS6-positive advancing CAFs after MFP treatment. **E.** WB reveals total and pAkt in Ser473, pS6, and ERK1/2. MFP induced time-dependent Akt and S6 activation. **F.** IF for F480, CD4 and CD8. **G.** IHC for Ki67 shows positive cells in tumor's periphery and center. Bar 100 μm.

To define the early events by which treatment-induced stromal activation of S6 leads to tumor regression, we assessed time-dependent response to MFP treatment as it had induced the most powerful effect. The tumor inhibitory effect (Figure 3A) was preceded by tissue rearrangements, as assessed by histology (Figure 3B) and immunostaining (Figure 3C). Cytostasis and apoptosis became evident in the epithelial compartment after 6 h of MFP (Figure 3A). The stromal reaction, including increased stromal area (Figure 3A, 3B), increased number of αSMA-positive CAFs (not shown), deposition of collagen-I (not shown), and increased number of CD31 foci (Figure 3C), was evident after 12 h of administration of MFP following a time-dependent manner. Labeling of blood vessels with fluorescent lectins (Figure 3C) confirmed that MFP induced functional new vessels.

Consistent with the results in Figure 2D, as C4-HD tumor regression progressed in response to MFP, the activation of PI3K/Akt/mTOR pathway was reduced in C4-HD tumor parenchyma and increased in tumor stroma (Figure 3D). Advancing CAFs are the stromal cells that stain for pS6 shortly after MFP therapy. The increase in stromal pS6-positive CAFswas time-dependent and elucidates the changes on pAKT and pS6 levels detected by western blot (Figure 3E). Thus, the increasing stromal component in MFP-treated tumors accounts for the increased overall pS6 levels.

We found that the rapid stromal reaction as assessed by increased CAFs and vasculogenesis was accompanied by immune cell infiltration. After 48 h of MFP administration, the intratumor presence of macrophages (F480+) and lymphocytes CD4+ and CD8+ was confirmed (Figure 3F). We also observed Ki67-positive stromal cells in the periphery and decreased Ki67 positivity in the center of the tumor, suggesting proliferative activation of tumor-associated stromal cells and/or migration and invasion of proliferative stromal cells from outside the tumor (Figure 3G). Together, the results show that the stromal reaction, hours after MFP administration, is associated with an increase in neovascularization and tumor cell infiltration.

**Figure 4:**
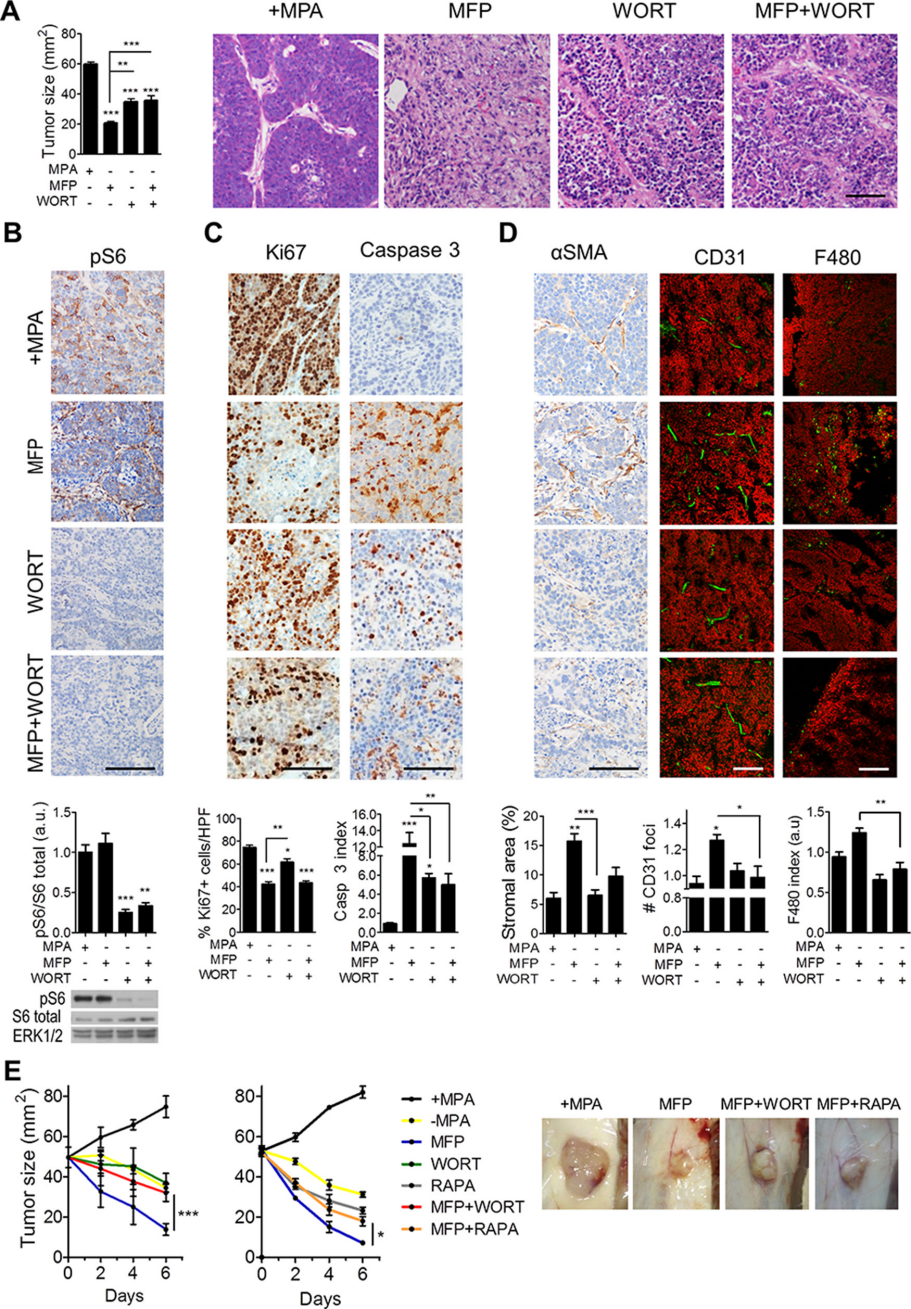
Inhibition of PI3K/Akt/mTOR pathway interferes with the stromal reaction induced by endocrine therapy in C4-HD tumors C4-HD tumors were treated for 48 h with MFP, WORT or the combination. **A. Left**: MFP-treated tumors showed smaller size compared to WORT or MFP+WORT-treated tumors. **Right**: H&E staining reveals greater tissue remodeling in MFP-treated tumors. **B. Top**: IHC for pS6 shows that WORT inhibited MFP-induced levels. **Bottom**: WB revealing total and pS6, and ERK1/2. **C.** IHC shows that after 12 h of treatment, WORT reduced Ki67 and induced caspase 3 to a lesser extent than MFP. **D.** IHC for αSMA and IF for CD31 and F480, show that MFP, but not WORT, affected stromal area, number of CD31 foci and macrophages infiltration. **E. Left**: C4-HD tumor sizes after 6 days of treatment with WORT or RAPA (5 mg/kg/day). Combination with WORT or RAPA significantly impaired MFP-induced regression. **Right**: Representative photographs of treated tumors. Bar: 100 μm.

To evaluate PI3K/Akt/mTOR pathway activation in the regression of C4-HD tumors, we used the PI3K inhibitor WORT alone or in combination with MFP for 48 h, finding that WORT interfered with MFP-induced tumor reduction and tissue remodeling (Figure 4A). At 12 h, WORT effectively inhibited the PI3K/Akt pathway in both parenchymal and stromal compartments as assessed by immunohistochemistry and western blot (Figure 4B). Strikingly, the combination of MFP and WORT reduced the apoptosis, tissue remodeling, neovascularization and immune cell infiltration caused by MFP (Figure 4C, 4D).

These results suggest that C4-HD tumor regression induced by MFP requires stromal activation of PI3K/Akt signaling and its inhibition interferes with the course of tumor regression. Extending the observation to six days of treatment revealed a sustained inhibition of MFP response when combined with WORT (Figure 4E). Further evidence that this was due to a requirement for PI3K/Akt signaling was provided by the observation that similar effects were found when the mTOR inhibitor rapamycin (RAPA) was combined with MFP (Figure 4E; Supplementary Figure S2). Although MFP+RAPA still induced necrosis, almost no stromal reaction was observed and reduction of tumor size was shorter than with MFP alone.

To verify that tumor regression specifically requires PI3K/Akt activation in the stroma, we evaluated the response of these treatments on C4-HD tumor cells growing on 3D Matrigel (Supplementary Figure S3A), finding again that MFP was the most effective treatment for decreasing Ki67 and increasing caspase 3 staining (Supplementary Figure S3B, S3C). However, in isolated C4-HD cells, WORT did not interfere with MFP-induced cytostasis or apoptosis (Supplementary Figure S3B, S3C). Thus, the unfavorable effect of WORT on endocrine-induced C4-HD tumor regression was not due to a direct effect on tumor cells, but rather due to its effect on the tumor stroma itself. These results demonstrate the existence of a critical stroma-dependent tumor regression mechanism that is suppressed when PI3K/Akt signaling is inhibited.

### The level of PI3K/Akt pathway activation in tumor cells determines the extent of stromal reaction as well as cell viability in response to therapy

**Figure 5:**
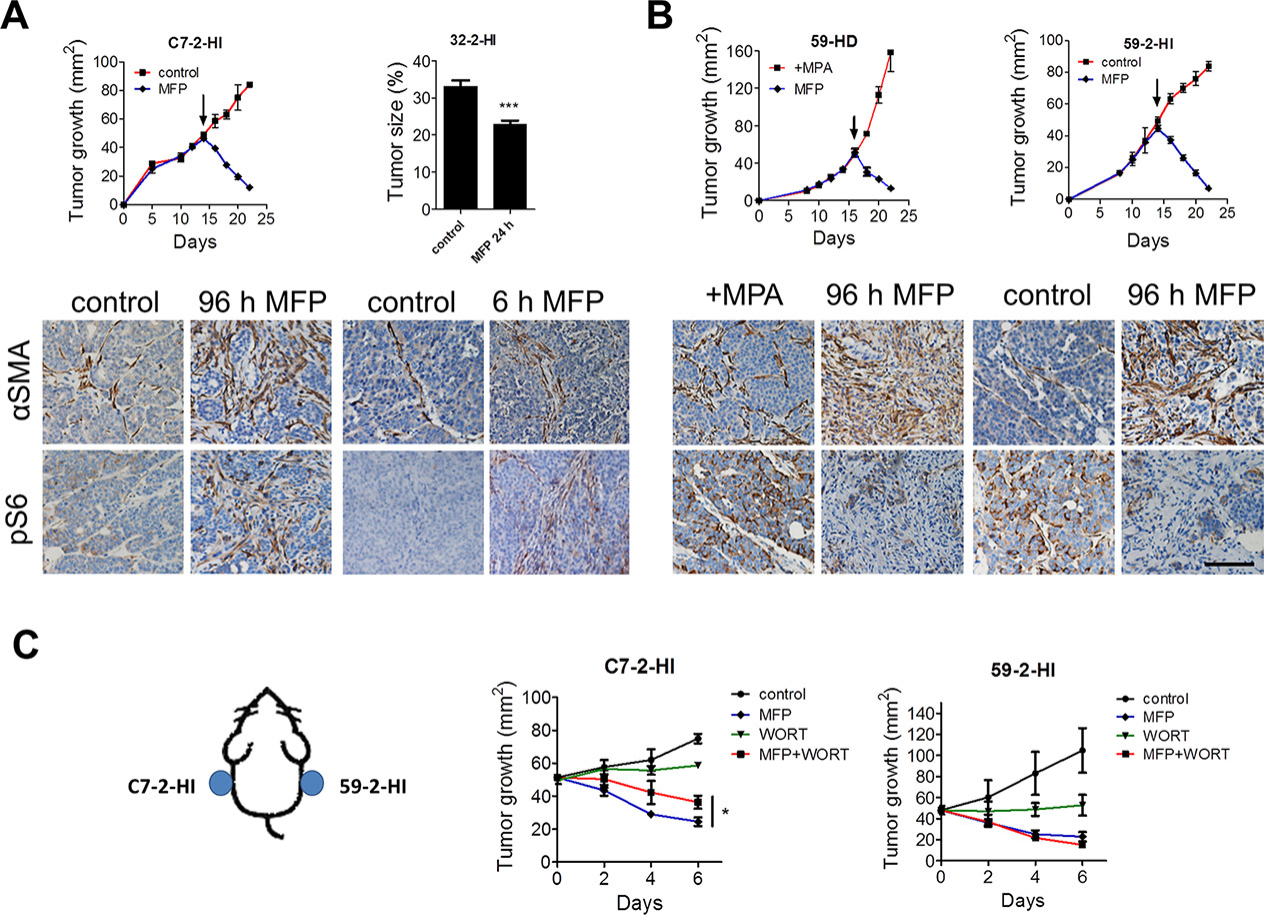
Tumor level of PI3K/Akt pathway determines the extent of stromal reaction in response to therapy **A & B.** When C7–2-HI, 32–2-HI, 59-HD or 59–2-HI tumor variants reached a size of 50 mm^2^ (arrows), mice were treated with MFP or saline solution (control). Tumors were treated for 6 days, except for 32–2-HI tumors that were treated for only 6 or 24 h since its response is extremely fast. **Top**: Growth curves show that MFP caused tumor regression. For 32–2-HI bar graph represents tumor size after 24 h of treatment. Bottom: IHC for αSMA and pS6 previous to MFP treatment shows that parenchymal pS6 levels were lower in C7–2-HI and 32–2-HI than in 59-HD and 59–2-HI tumors. After MFP treatment, pS6 increased in the stromal compartment in C7–2-HI and 32–2-HI tumors, and it reduced in the parenchymal compartment in 59-HD and 59–2-HI tumors. **C. Left**: C7–2-HI and 59–2-HI tumors were transplanted simultaneously in the same mouse in opposite flanks and when tumors reached a size of 50 mm2, treatments started (day 0) with MFP, WORT or the combination, and continued for 6 days. **Right**: Tumor sizes show that C7–2-HI tumors responded poorly to WORT whereas 59–2-HI tumors reduced tumor size after WORT treatment. Bar: 100 μm.

Therapy-induced stromal pS6 was observed in C4-HD (Figure 2D) but not in C4-HI (Figure 1D) tumors. To determine the effect of intrinsic tumor heterogeneity on the stroma-dependent tumor regression process, we evaluated other variants of the MPA-induced model. In tumor variants that show low levels of parenchymal pS6 when grown as allografts (Figure 5A), MFP-induced tumor reduction involves stromal remodeling, αSMA expression and pS6 increase, and MFP-induced tumor regression was impaired by WORT (Figure 5C). By contrast, in tumor variants that display high levels of parenchymal pS6 when grown as allografts (Figure 5B), MFP-induced tumor reduction involves stromal remodeling with αSMA expression, decreased parenchymal pS6, and little stromal pS6. In this case, tumor regression was enhanced by the combination of MFP and WORT (Figure 5C). These results indicate that the differential response to MFP is related to basal PI3K/Akt and pS6 levels rather than to MPA-dependent status.

**Figure 6:**
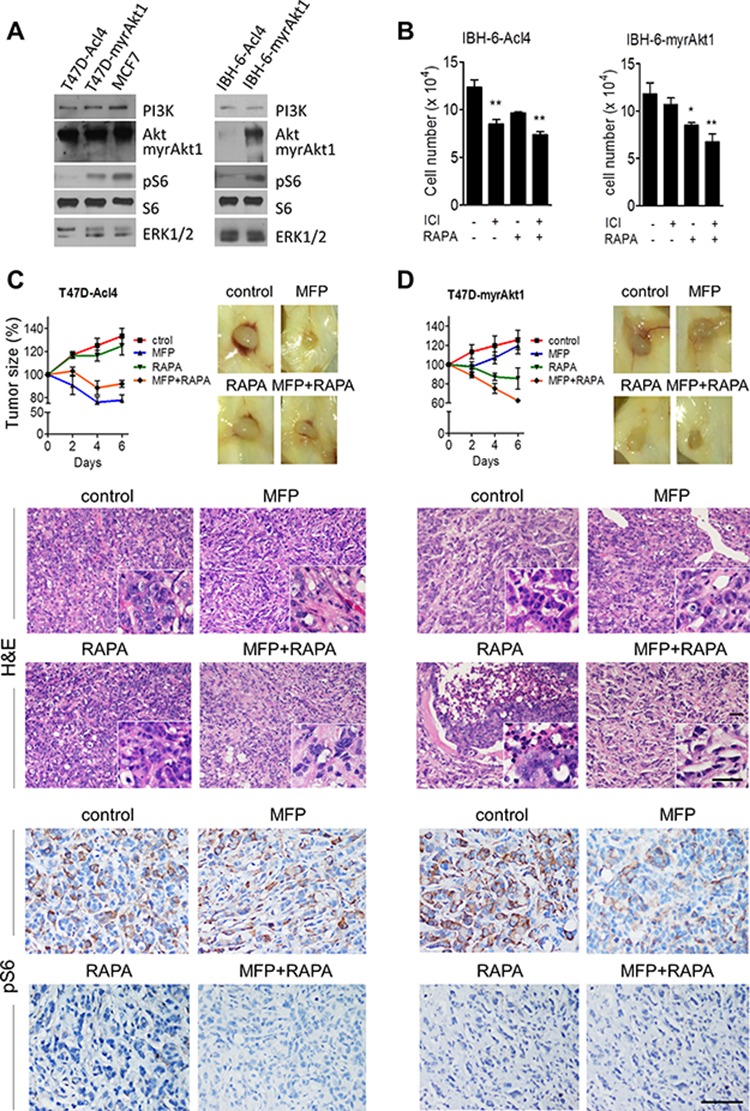
Tumor cell level of PI3K/Akt pathway determines response to therapy **A.** WB from cellular extracts revealing PI3K, Akt, total and pS6, and ERK1/2. Akt antibody detected both the endogenous and the myristoylated deleted myrAkt1variant. MCF-7 cells were used as a control. **B.** IBH-6-Acl4 and IBH-6-myrAkt1 cells were treated with ICI 1 μM, RAPA 0.1 μM or the combination for 6 days, and final cell number was counted. **C&D.** T47D-Acl4 and T47D-myrAkt1 cells were injected into NOD/SCID mice. Tumors of 30 mm^2^ were treated with MFP, RAPA (5 mg/kg/day) or the combination, and continued for 6 days. **Top, left**: Tumor sizes expressed as % of pretreatment size. T47D-Acl4 tumors responded mainly to MFP whereas T47D-myrAkt1 tumors responded mainly to RAPA. Representative photographs (**right**) and H&E (**center**) of control and treated tumors. **Inserts**: T47D-Acl4 tumors show MFP-induced blood vessels. **Bottom**: IHC for pS6 shows that stromal levels were higher in T47D-Acl4 than in T47D-myrAkt1 tumors after MFP treatment. Bar: 50 μm.

To assess the dependence of the stromal response on endocrine targeting tumor reduction in human breast cancer cell models, we employed T47D and IBH-6 cells expressing vector only (Acl4) or expressing an activated variant of Akt1 (myristoylated Akt1, myrAkt1) (Figure 6A). We used RAPA to overcome the constitutive activation of Akt1 due to the myrAkt1 plasmid. In proliferation assays using the IBH-6 cell series, the antiestrogen ICI showed a stronger inhibition of proliferation than RAPA in vector control cells, whereas RAPA was more effective than ICI in Akt1-expressing cells (Figure 6B). We have previously shown that MFP inhibits T47D wild type xenograft growth [29, 30]. Here we found that orthotopic xenografts of T47D-Acl4 cells showed significant therapeutic response to MFP or MFP+RAPA but not to RAPA itself (Figure 6C), while xenografts of T47D-myrAkt1 cells were strongly inhibited by RAPA alone or RAPA combined with MFP, but not with MFP alone (Figure 6D). In T47D-Acl4 tumors, MFP induced strong tissue remodeling and neovascularization associated with tumor shrinkage (Figure 6C). By contrast, in T47D-myrAkt1 tumors, RAPA was required for induction of necrosis and tumor shrinkage (Figure 6D), while MFP did not induce tissue remodeling. As expected, pS6 levels were higher in T47D-myrAkt1 (Figure 6D) than in T47D-Acl4 tumors (Figure 6C) and were severely diminished by RAPA. Finally, pS6-positive CAFs were induced by MFP in T47D-Acl4 but not in T47D-myrAkt1 tumors (Figure 6C, 6D).

Together, our findings demonstrate that activation of S6 in tumor cells is prognostic of therapeutic response in diverse models of breast cancer and represents a potential biomarker to distinguish for which tumors combination of PI3K/Akt/mTOR inhibitors with anti-endocrine therapies would be beneficial, and for which they could have the potential to be detrimental.

### Correlation between stromal pS6 and αSMA suggests a modulatory effect of PI3K/Akt pathway in the therapeutic response of human breast cancer

**Figure 7:**
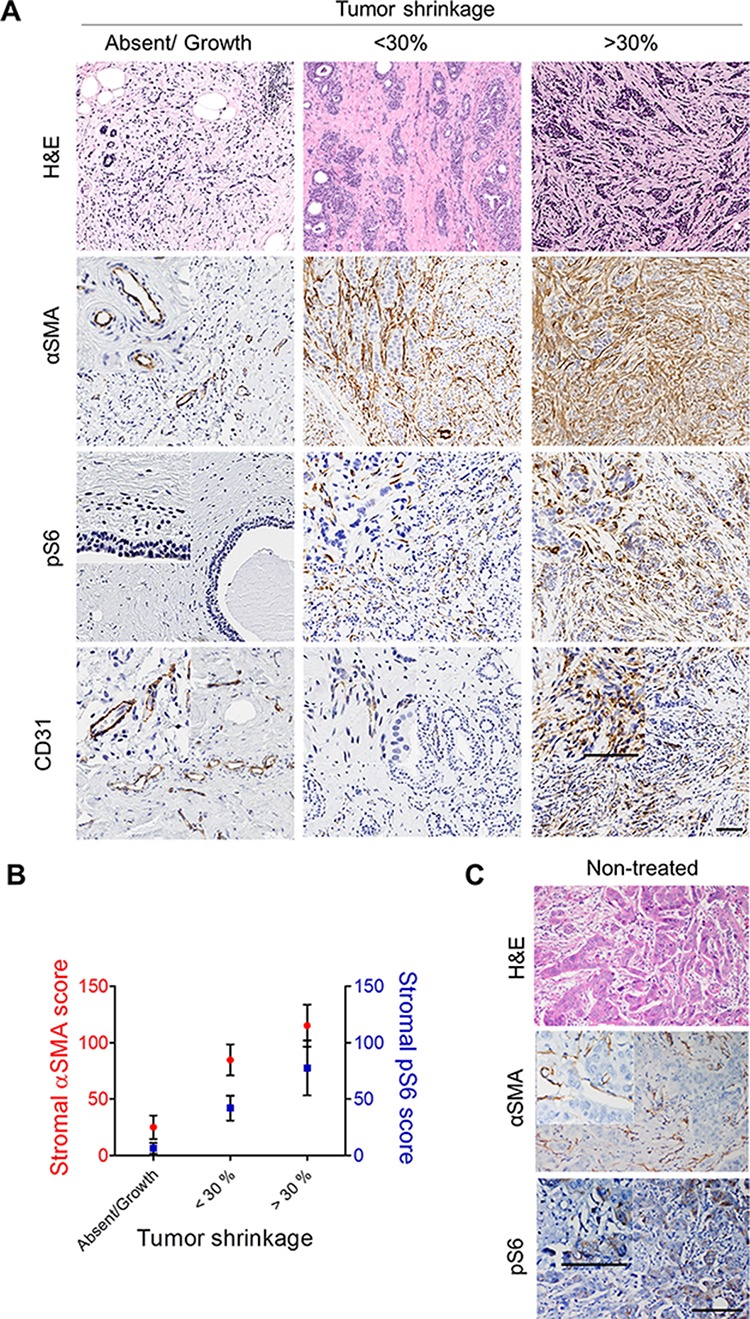
αSMA correlates with tumor reduction and stromal pS6 after neoadjuvant endocrine therapy Breast tumor samples were classified according to the percentage of tumor reduction, between no response (increase or no change in tumor mass) *n* = 4, intermediate response (less than 30% reduction) *n* = 12, or better response (more than 30% reduction) *n* = 8. **A.** H&E and IHC for αSMA, pS6 and CD31 in one representative tumor of each group. The amount and intensity of αSMA and stromal pS6 label increased according to % of tumor reduction. **Inserts**: αSMA, pS6 and CD31 were mainly localized in active areas of advancing stroma. **B.** The entire cohort of 24 patients was distributed for the graph in terms of tumor reduction with the arbitrary cut off of 30% and analyzed as a whole for correlation between the three parameters. Stromal αSMA correlated significantly with stromal pS6 score (*p* = 0.039, Spearman Rho) and with the % of tumor reduction (*p* = 0.036, Spearman Rho). **C.** H&E and IHC for αSMA and pS6 in tumor areas of one representative non-treated patient, showing the staining of pS6 in the parenchyma and its absence in the stroma. Bar: 100 μm.

To assess how these pathways impact human breast cancer treatment response, we evaluated breast tumor samples from a set of 24 women who received neoadjuvant endocrine therapy at the Mayo Clinic (cohort characteristics in Table 1). We analyzed the type and grade of stromal reaction in the post-treatment samples, assessing collagen-I (not shown), αSMA, CD31, and pS6 score in the parenchyma and in the stroma (Figure 7A). To evaluate the therapeutic response, we compared tumor size before starting therapy and immediately before surgery. The clinical response to treatment correlated significantly with stromal αSMA (Figure 7B). We also found that while αSMA, pS6 and CD31 were associated in areas where the stroma increased and the tumor regressed (Figure 7A inserts), CD31 levels were not significantly correlated with αSMA or pS6 scores in the overall sample. Strikingly, stromal but not parenchymal pS6 correlated significantly with αSMA (Figure 7B) and in quiescent or fibrotic stroma pS6 was low or absent (Figure 7A). Furthermore, actively regressive tumor areas showed that αSMA, pS6 and CD31 were mainly localized in the advancing stroma (Figure 7A inserts). While these results are somewhat limited by considerations of sample size and heterogeneity of patient treatment regimens, our findings are supportive of our model that tumor regressive processes in humans also involve stromal PI3K/Akt/mTOR pathway activation.

**Table 1:** Patient characteristics for treated-breast carcinomas from Mayo Clinic

Patient #	Age (years)	Tumor size (cm)		Node	Treatment	Histopathological data and prognostic factors					
		Pre-treatment	Post-treatment		Drug	Days	Subtype	Grade	ER	PR	HER-2
1	83	5, 1	7, 5	-	anastrozol	153	ibc NST	moderate	+	+	-
2	66	7, 1	7	-	anastrozol	156	ibc NST	moderate	+	+	-
3	59	4	2, 9	+	exemestane	125	ibc NST	moderate	+	+	-
4	57	8	8, 8	+	anastrozol	123	ibc NST	high	+	+	-
5	67	10	4, 5	+	anastrozol	163	ibc NST	moderate	+	+	+
6	82	2, 4	1, 8	-	letrozol	43	ibc NST	moderate	+	+	-
7	71	2, 3	1, 9	-	anastrozol	120	ibc NST	moderate	+	+	-
8	70	n/a	12, 5	+	letrozol	347	ibc NST	moderate	+	+	-
9	64	2, 5	1, 7	+	letrozol	205	ibc NST	moderate	+	+	-
10	83	7	6, 8	+	anastrozol	251	ibc NST	moderate	+	+	-
11	57	6, 2	8, 1	+	anastrozol	157	ilc	moderate	+	n/a	-
12	54	5, 2	4, 8	+	exemestane + goserelin	281	dcis	low	+	+	-
13	67	5, 2	3, 8	+	letrozol	166	ibc NST	high	+	+	n/a
14	70	2, 4	1, 5	-	anastrozol	105	ibc NST	moderate	+	+	-
15	57	10	4, 3	+	anastrozol	273	ibc NST	moderate	+	+	-
16	75	2, 5	2, 3	+	letrozol	119	ibc NST	moderate	+	+	-
17	77	n/a	2, 4	-	anastrozol	503	ibc NST	moderate	+	+	n/a
18	95	1, 6	1, 7	+	tamoxifen	98	dcis	moderate	+	+	-
19	76	4, 2	1, 8	-	anastrozol	56	ibc NST	low	+	+	n/a
20	77	4	3	+	tamoxifen	107	ibc NST	low	+	+	n/a
21	62	6, 2	5, 8	+	anastrozol	77	ibc NST	moderate	+	+	-
22	72	4, 9	0, 8	-	anastrozol	155	ibc NST	low	+	+	-
23	68	8	7, 2	-	anastrozol	97	ibc NST	moderate	+	+	-
24	65	3	2, 5	+	anastrozol	126	ibc NST	moderate	+	+	-

*dcis: ductal carcinoma *in situ*, Ibc NST: invasible breast carcinoma of not special type, ilc: invasive lobular carcinoma, n/a: data not available

In another set of 18 human samples naïve of any treatment (cohort characteristics described in Table 2), αSMA was present in fibroblasts, myoepithelial cells and vascular structures (Figure 7C). Epithelial neoplastic cells showed pS6-positive staining in 13 of 18 samples (65%), but pS6 was totally absent in the stroma in these samples. These findings support our model that stromal pS6 discriminates, at an early stage of treatment, which subset of patients has activated PI3K/Akt pathway and could therefore benefit from an anti-endocrine/anti-PI3K/Akt combination therapy.

**Table 2:** Patient characteristics for non-treated breast carcinomas from CORI Cancer Center

Patient #	Age (years)	Tumor size (cm)	Node	Histopathological data and prognostic factors					
				Subtype	Grade	ER (%)	PR (%)	HER-2	Ki-67 (%)
1	42	2	+	ibc NST	moderate	80	40	-	40
2	n/a	2	+	ibc NST	low	90	20	-	20
3	72	2, 5	-	ibc NST	high	80	10	-	10
4	78	n/a	n/a	ibc NST	high	70	10	-	10
5	n/a	1	n/a	papilary carcinoma	n/a	90	30	-	30
6	n/a	2	n/a	ibc NST	moderate	90	30	-	30
7	64	2	n/a	ibc NST	moderate	80	60	-	60
8	n/a	1, 3	n/a	ibc NST	moderate	30	2	-	2
9	36	n/a	-	papilary carcinoma	high	80	20	-	20
10	59	0, 4	n/a	ibc NST	moderate	90	30	-	30
11	n/a	1, 7	n/a	ibc NST	high	90	30	-	30
12	45	1, 7	n/a	ibc NST	high	70	5	-	5
13	n/a	n/a	n/a	apocrine carcinoma	high	70	5	-	5
14	73	n/a	n/a	ibc NST	high	90	10	-	10
15	n/a	2	n/a	mucinous carcinoma	low	100	60	-	60
16	66	n/a	+	ibc NST	moderate	90	5	-	5
17	n/a	2, 3	+	ibc NST	moderate	90	5	-	5
18	63	1, 5	-	ibc NST	moderate	30	60	-	60

*ibc NST: invasive breast carcinoma of not special type, n/a: data not available.

*% (positive cells/total number of cells)

## DISCUSSION

Here we defined a novel role of the stromal reaction in breast tumor regression after anti-endocrine therapy. We found that stromal expression of pS6 may represent a biomarker for the benefit of incorporating PI3K/Akt/mTOR inhibitors into neoadjuvant endocrine therapies. Using mouse models of ER/PR-positive breast carcinomas, orthotopic xenografts of T47D human breast cancer cells, and clinical samples from breast cancer patients undergoing neoadjuvant endocrine therapy, we found that the intrinsic tumor heterogeneity for PI3K/Akt/mTOR activation as assessed by S6 protein phosphorylation is associated with therapeutic response. Stromal pS6 is present exclusively after therapy and is absent in non-treated tumors or in endocrine-treated tumors with a poor reactive stroma. We suspect that the presence of pS6 in stromal or in parenchymal compartments after neoadjuvant endocrine therapy is related to particular mechanisms involved in tumor regression. Thus, evaluation of parenchymal and stromal expression of pS6 early in treatment may identify which patients would benefit from combining PI3K/mTOR inhibitors with neoadjuvant endocrine therapies, and more critically, for which patients this combination would be potentially detrimental. Future studies are necessary to validate this hypothesis and its possible relevance for selecting patients for PI3K/Akt/mTOR pathway-targeting drugs in clinical practice.

In both pre-clinical and clinical settings, we found that the stromal reaction associated with activation of αSMA and deposition of collagen-I is linked to better therapeutic outcomes. We further found that in the mouse model, MFP-induced formation of functional new vessels relates to tumor cell infiltration of pS6-positive CAFs and immune cells, likely representing a crucial role of stromal pS6 in the remodeling and healing processes that take place during early stages of tumor regression. Previous reports have suggested that human breast CAFs can directly promote angiogenesis through the expression of stromal cell-derived factors [31] and that CAFs promoted macrophage recruitment, neovascularization, and tumor growth in a mouse model of squamous skin carcinogenesis [32]. We speculate that mesenchymal fibroblasts could be the key cell type responsible for the tumor regressive stromal reaction, but further studies are necessary to fully evaluate this hypothesis. We found that in tumor variants in which there is low intrinsic tumor level of PI3K/Akt/mTOR relative to that found in the stroma, blockage of pS6 activation through the use of PI3K/mTOR inhibitors interferes with MFP-induced neovascularization, immune cell infiltration and tumor regression. However, in tumor variants with a high intrinsic tumor level of PI3K/Akt/mTOR, MFP as a single agent did not induce tumor regression, and its combination with PI3K/mTOR inhibitors is required to induce a therapeutic response. Since in human samples we found that actively regressive tumor areas of advancing stroma stained positive for αSMA, pS6 and CD31, our results in the mouse model could provide mechanistic insight into how stromal pS6 activation occurs and how it contributes to breast tumor regression after endocrine therapy.

Variations in breast cancer-associated stromal histology have been described from predominantly cellular (fibroblasts/myofibroblasts) to fibrillar (dense collagenous stroma) subtypes [33]. Such stromal variations are associated with differential paracrine activation by growth factors and cytokines. Recent studies have evaluated variations in tumor stroma as a predictor of outcome in breast cancer patients [34], finding that a higher ratio of tumor to stroma is associated with recurrence and poorer long-term survival. Another recent study evaluating tumor stroma after endocrine therapy in breast cancer [35] found that molecular differences between invasive lobular cancer (ILC) and infiltrating ductal carcinomas (IDCs) are maintained following endocrine treatment. It is becoming clear that detailed analyses of tumor parenchyma and stroma, as well as their interactions will be required to develop therapies that target the tumor microenvironment [21, 36–40]. In this context, our results have particular relevance by providing insight into stromal biomarkers of antitumor efficacy that are evident soon after treatment begins, which is lacking in current therapeutic protocols. The parameters that we identified here (αSMA and pS6) are easily quantified by pathological examination using normal clinical procedures.

While endocrine therapies can be initially effective for ER/PR breast cancer patients, acquired or *de novo* resistance and subsequent recurrence remain significant clinical problems. Pre-clinical *in vivo* studies have recently been developed [41, 42] and an improved understanding of the interaction of endocrine and PI3K/Akt/mTOR inhibitors in neoadjuvant settings is necessary to break down the heterogeneity in responses to target therapy as reported in the clinic [13]. We assessed model systems and human breast tumor samples to dissect how stromal activation of PI3K/Akt affects response to endocrine therapies. Our findings demonstrate that activation level of S6 in tumor cells is prognostic of therapeutic response and could be relevant to explore the involvement of PI3K/Akt/mTOR targeting therapy to avoid or delay hormone independence and consequently endocrine resistance. The molecular mechanisms that contribute to tumor regression after therapy, conferring the response of the tumor cells to MFP and the induction of S6 phosphorylation in the stromal cells, remain to be defined. The authors speculate that these mechanisms relate more with a wound healing process than to tumor growth events. Further experiments are being performed to examine the molecular interactions between tumor cells and stromal cells during tumor regression after therapy. Also, longer-term studies will be necessary to determine if the more effective methods for inducing tumor regression identified in our study also confer reduced rates of tumor relapse.

It has been proposed that tumors with mutations at the catalytic p110α subunit of PI3K (*PIK3CA* mutations) that may confer activation of the PI3K/Akt/mTOR pathway are more sensitive to PI3K/mTOR inhibitors [43], although the prognostic value of PIK3CA mutations in ER-positive breast cancer is still controversial [44–47]. The effect of PI3K/mTOR inhibitors has yet to be validated through reliable biomarkers of efficacy [48]. Phosphorylated S6 and its kinase p70S6K also have been proposed to predict tamoxifen resistance [49]. The striking finding in our pre-clinical models, supported by our results with human breast cancer biopsies, is that pS6 is highly expressed in invading and reactive stroma after therapy. It has been reported that stromal pS6 increased in the fibroblasts embedded within the tumors in Caveolin-1 knock out mice [50] and the authors related that finding with angiogenesis and with breast tumor hormone-independent growth. The authors also reported these effects can be reduced by RAPA and suggested the involvement of the stromal mTOR pathway on blood vessel formation and tumor growth in Caveolin-1–deficient tumors. Strikingly, here we propose that the proliferative stroma with activated mTOR signaling could also be a good prognostic indicator of the tumor regressive process in a particular tumor context. Then, pS6 is a potential early biomarker that could predict better clinical outcome after endocrine therapy in those tumors with a high percentage of stained stromal cells. On the basis of the results present here, we speculate that tumor level and localization of PI3K/Akt/mTOR pathway activation before neo/adjuvant therapy can be used to predict which patients will benefit with a combination therapy with PI3K/mTOR inhibitors and which patients will not. For those in the latter category, it may be that endocrine therapy only should be recommended.

An increase in microvasculature and tumor infiltration by MFP was recently reported in other MPA-induced tumors as well as in T47D xenografts [30]. Our results are consistent with those reported by Nakasone et al., who found that the microenvironment contributes critically to the response to chemotherapy via regulation of vascular permeability and immune cell infiltration [51]. Furthermore, we show here that WORT interferes or potentiates the MFP-induced neovasculature in C4-HD or C4-HI tumors, respectively. This dual effect of PI3K in angiogenesis was already observed both in normal tissues and in cancers [52, 53]. Furthermore, it has been reported in mice that inhibition of PI3K could normalize vascular structure and increased perfusion of breast tumors improving delivery of doxorubicin [54]. Even though the mechanisms by how the stromal activation interact with endocrine therapies remains to be demonstrated, we speculate that in some type of tumors pS6 stromal activation could drive stromal reaction involving angiogenesis that contributes to tumor infiltration of immune cells and also to tumor shrinkage through improvement of drug delivery. Further studies will be necessary to assess this possibility.

A limitation of the MPA-induced murine model used here is that it is a progesterone-driven model. However, endocrine sensitivity or resistance coexists for both antiprogestins and antiestrogens [25], supporting the use of this model as appropriate for studying ER/PR-positive disease. Another limitation of our study is that we used ER/PR-positive T47D cells that have oncogenic mutations in PIK3CA [55]. Nevertheless, introduction of myrAkt1 construct in T47D cells still increases PI3K/Akt/mTOR pathway. Referring to human samples, different endocrine therapies have been used in the cohort of 24 patients available for this study. Considering that each therapy could have different mechanisms of actions and resistance, a more comprehensive study that include patients from multiple clinical centers could provide a more complete assessment. Furthermore, studies considering clinical, histopathological and molecular parameters should be done in biopsies before and after treatment to validate the potential role of pS6 in the prediction of therapy efficacy.

## CONCLUSIONS

Our findings demonstrate that the stromal reaction accompanied by αSMA increase that occurs early in tumor regression resembles a healing process that is indicative of overall therapeutic response. We find that stromal and parenchymal phosphorylated ribosomal protein S6 (pS6) downstream PI3K/Akt/mTOR pathway constitutes potential biomarkers of targeting therapy efficiency. Moreover, we show that PI3K/mTOR inhibition in combination with endocrine targeting therapies represent a useful approach only in tumors that have high levels of PI3K/Akt/mTOR in the parenchymal compartment. The current challenge will be to evaluate the generalizability of pS6 levels and localization as biomarkers to design, guide and monitor treatment strategies for breast cancer patients.

## MATERIALS AND METHODS

### Animals

Two-month-old virgin female BALB/c mice from the University of La Plata and NOD/LtSz-scid/IL-2Rgamma null mice (NSG) from The Jackson Laboratory were bread at IByME (Animal Facility). Animal care and manipulation were in agreement with the International Guide for the Care and Use of Laboratory Animals [56].

### Tumors from MPA-induced model of breast cancer

The MPA murine model of breast cancer was formerly established in BALB/c mice reviewed in [25]. For details about drugs and treatments see Supplementary Material 1.

### Cell lines and establishment of tumor xenografts

IBH-6 [28, 57] and T47D (ATCC) human breast cancer cells were stably transfected with an empty Acl4 retroviral vector, or a myristoylated human Akt1-Δ4–129 (myrAkt1) construct, as described in [28]. Cell counting and xenograft implantation were previously described [29, 30], for more details see Supplementary Material 2.

### Human breast cancer tissue samples

Human samples were obtained from a tissue bank with no selection criteria. The study on treated samples was limitated to the number of available samples of breast carcinomas that had been treated with neoadjuvant endocrine therapy and undergone surgical resection at Mayo Clinic. Written informed consents were received from participants prior to inclusion in the study and are available in Mayo Clinic and CORI Cancer Center. Confidentiality was preserved for the investigators in a codified form. Mayo Clinic provided approval for this research through its Institutional Review Board. The IByME Ethics Committee approved the study with CORI Cancer Center.

### Histopathological analysis

Tumors were fixed, paraffin-embedded (FFPE) and sectioned. The histopathological features of both tumor parenchyma and stroma were evaluated in hematoxylin and eosin (H&E)-stained slides. Apoptosis, mitosis, necrotic and stromal area quantification are described in Supplementary Material 3.

### Immunohistochemistry (IHC)

FFPE tissues were stained for primary antibodies. For details about IHC protocol, Ki67 and caspase 3 quantification see Supplementary Material 4.

### Immunofluorescence (IF)

Tumor sections were analyzed under a Nikon Eclipse E800 Confocal Microscope. For details see Supplementary Material 5.

Epithelial cell clusters were obtained by differential sedimentation from C4-HD and C4-HI tumors as previously described [27]. For details about primary cultures, IF protocol and indexes quantification see Supplementary Material 6.

### Western blot (WB)

For details about protocol see Supplementary Material 7.

### Statistical analyses

Statistical analyses were performed with the GraphPad Prism™ software 5.0 (GraphPad Software, Inc.). One way ANOVA followed by Tukey multiple post *t*-test were used to compare means of multiple samples. When comparing the means of two different groups, two-sided Student's *t*-test was applied. Tumor growth curves were studied using regression analysis, and the slopes compared using analysis of variance. Data from patient samples was analyzed using SSPN software (IBM) to find correlations using Spearman's Rho test. In all graphs, the mean ± SEM are shown. Significant differences *, *p* < 0.05; **, *p* < 0.01; ***, *p* < 0.001.

## SUPPLEMENTARY MATERIAL FIGURES


