# Correction: Activation of PI3K/Akt/mTOR signaling in the tumor stroma drives endocrine therapy-dependent breast tumor regression

**DOI:** 10.18632/oncotarget.28728

**Published:** 2025-05-20

**Authors:** María Laura Polo, Marina Riggio, María May, María Jimena Rodríguez, María Cecilia Perrone, Melody Stallings-Mann, Diego Kaen, Marlene Frost, Matthew Goetz, Judy Boughey, Claudia Lanari, Derek Radisky, Virginia Novaro

**Affiliations:** ^1^Instituto de Biología y Medicina Experimental, Protein Kinases and Cancer Laboratory, Buenos Aires, Argentina; ^2^Mayo Clinic Comprehensive Cancer Center, Department of Cancer Biology, Jacksonville, FL, USA; ^3^Centro Oncológico Riojano Integral, Medical Oncology, La Rioja, Argentina; ^4^Department of Medical Oncology, Mayo Clinic, Rochester, MN, USA; ^5^Department of Surgery, Mayo Clinic, Rochester, MN, USA

**This article has been corrected:** The authors discovered duplication errors in Figure 4 and Figure 6. Specifically, in Figure 4B top panel, tumor images stained with pS6 and representing WORT and MFP+WORT-treated tumors were found to overlap. The authors have replaced this panel with new images of the pS6 stained samples from the original experiment.

In Figure 6A there was a duplication of S6 WB images due to an incorrectly labeled picture for T47D cells. The authors have corrected this by using one of two additional sets of T47D WBs from the original experiment and replacing all panels for T47D cells. Uncropped images of two sets of T47D WBs from the original experiments have been provided.

In Figure 6D, an inadvertent duplication of IHC images for pS6 in T47D-myrAkt1 tumors treated with RAPA or RAPA+MFP (bottom right images) was present. The immunostaining was repeated using the same paraffin-embedded tissues from the original experiment, and unmodified data has been provided.

The authors have stated that these corrections do not alter the article’s conclusions. They sincerely apologize for any inconvenience caused to the readers.

Original article: Oncotarget. 2015; 6:22081–22097. 22081-22097. https://doi.org/10.18632/oncotarget.4203

**Figure 4:**
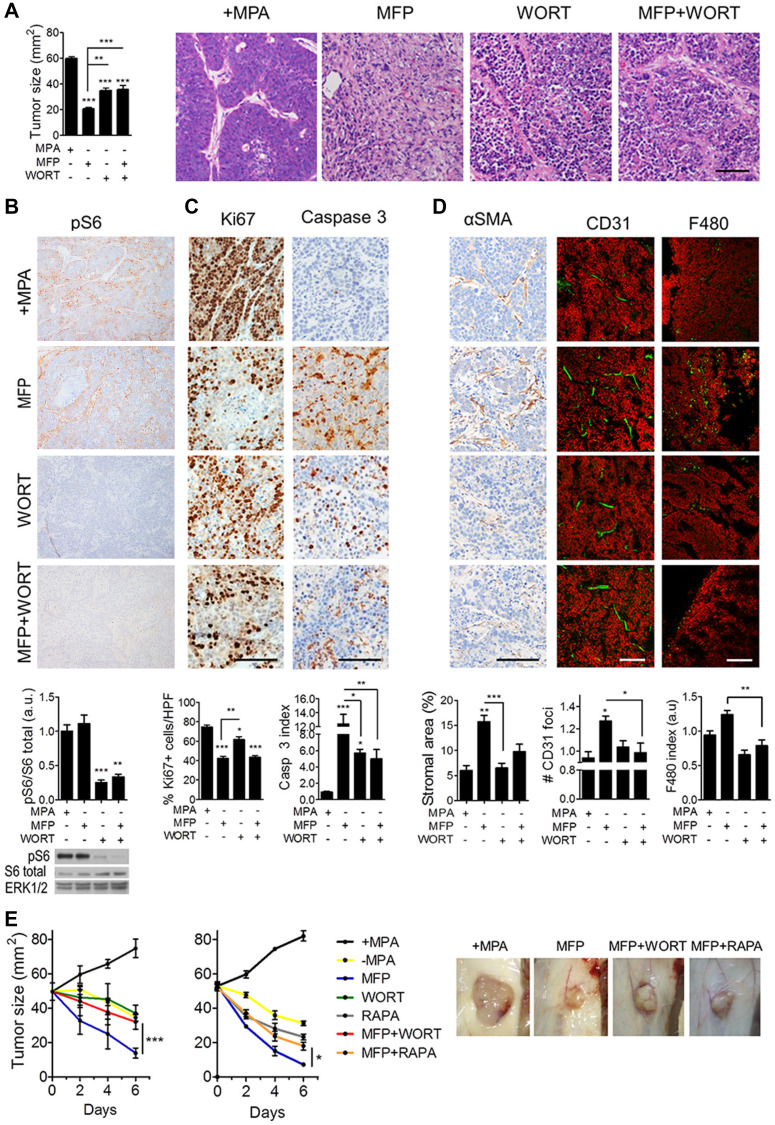
Inhibition of PI3K/Akt/mTOR pathway interferes with the stromal reaction induced by endocrine therapy in C4-HD tumors. C4-HD tumors were treated for 48 h with MFP, WORT or the combination. (**A**) Left: MFP-treated tumors showed smaller size compared to WORT or MFP+WORT-treated tumors. Right: H&E staining reveals greater tissue remodeling in MFP-treated tumors. (**B**) Top: IHC for pS6 shows that WORT inhibited MFP-induced levels. Bottom: WB revealing total and pS6, and ERK1/2. (**C**) IHC shows that after 12 h of treatment, WORT reduced Ki67 and induced caspase 3 to a lesser extent than MFP. (**D**) IHC for αSMA and IF for CD31 and F480, show that MFP, but not WORT, affected stromal area, number of CD31 foci and macrophages infiltration. (**E**) Left: C4-HD tumor sizes after 6 days of treatment with WORT or RAPA (5 mg/kg/day). Combination with WORT or RAPA significantly impaired MFP-induced regression. Right: Representative photographs of treated tumors. Bar: 100 μm.

**Figure 6:**
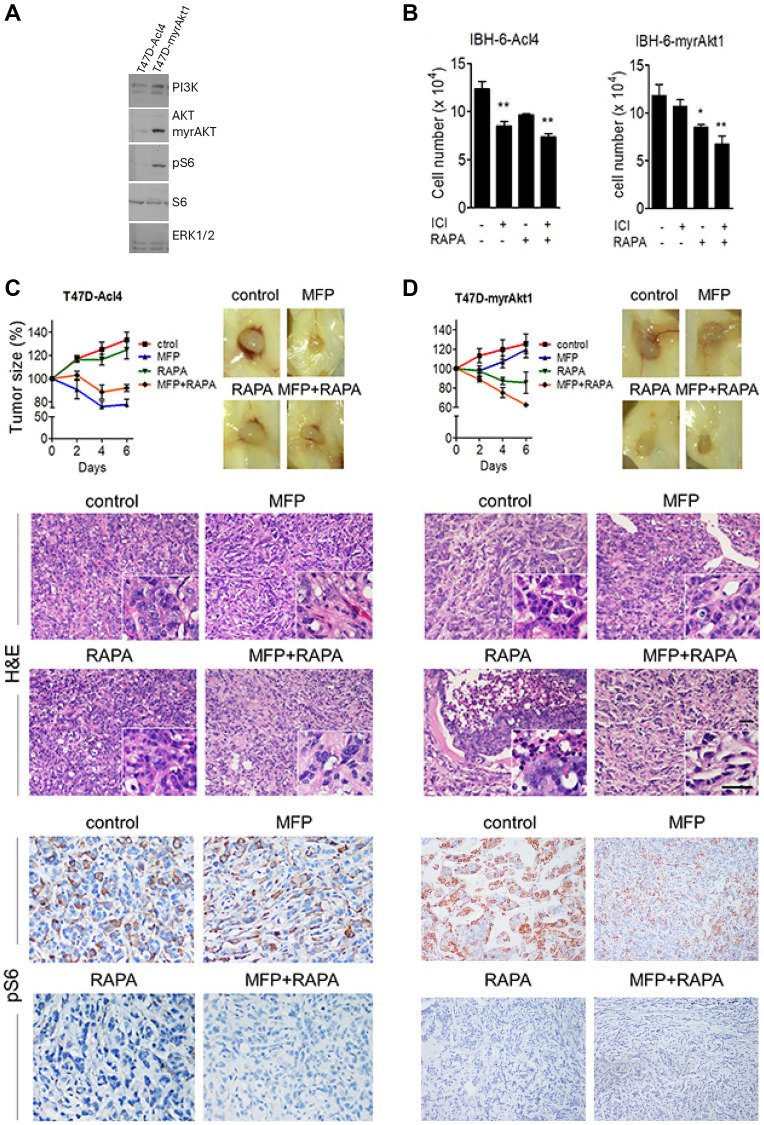
Tumor cell level of PI3K/Akt pathway determines response to therapy. (**A**) WB from cellular extracts revealing PI3K, Akt, total and pS6, and ERK1/2. Akt antibody detected both the endogenous and the myristoylated deleted myrAkt1variant. MCF-7 cells were used as a control. (**B**) IBH-6-Acl4 and IBH-6-myrAkt1 cells were treated with ICI 1 μM, RAPA 0.1 μM or the combination for 6 days, and final cell number was counted. (**C**, **D**) T47D-Acl4 and T47D-myrAkt1 cells were injected into NOD/SCID mice. Tumors of 30 mm2 were treated with MFP, RAPA (5 mg/kg/day) or the combination, and continued for 6 days. Top, left: Tumor sizes expressed as % of pretreatment size. T47D-Acl4 tumors responded mainly to MFP whereas T47D-myrAkt1 tumors responded mainly to RAPA. Representative photographs (right) and H&E (center) of control and treated tumors. Inserts: T47D-Acl4 tumors show MFP-induced blood vessels. Bottom: IHC for pS6 shows that stromal levels were higher in T47D-Acl4 than in T47D-myrAkt1 tumors after MFP treatment. Bar: 50 μm.

