# Supplementary material for: Activation of PI3K/Akt/mTOR signaling in the tumor stroma drives endocrine therapy-dependent breast tumor regression
Source: Oncotarget. 2015 Jun 8;6(26):22081–97. doi: 10.18632/oncotarget.4203 (PMC4673148; doi:10.18632/oncotarget.4203)
Supplement: Supplementary file 1 [file oncotarget-06-22081-s001.pdf]

SUPPLEMENTARY MATERIAL

Study design

The purpose of our study was to analyze mechanisms of tumor regression after short-term endocrine therapy, and to explore the effects of combinning endocrine agents with PI3K/Akt/mTOR inhibitors in breast cancer. We studied both parenchyma and stroma response to antitumor treatments using preclinical models of mammary carcinomas and patient samples. Pre-specified hypotheses were that variations in stromal gene activation would be associated with pathological and clinical response to anti-endocrine therapies in animal models and in human tissue biopsies. These hypotheses did not change upon data analysis.

In vivo animal experiments were performed at least twice using four mice per experimental group in each replica. Each figure represents one of the replicas. The number of mice required for the experiments was estimated according to previous results and pilot tests that allows a 25% difference between the means of control and treatment groups and a p value of 0.05. Animals were randomly and equally assigned to either control or treatment groups, and experiments were conducted in a controlled and non-blinded manner. Culture studies were performed three times and at least in duplicate for each experiment. Primary endpoints in animal studies were prospectively selected. In the MPA-induced model the studies involved short-term treatments to include the initiation of the tumor regressive process according to previous results. In xenograft studies the endpoint was selected to have divergence between therapeutic responses according to previous results, but with enough remnant tumor tissue to perform histological and protein examination. Animal care and manipulation were in agreement with the International Guide for the Care and Use of Laboratory Animals. The local ethics committee (IBYME-CE 027–2012 and 023–2014) approved the animal protocol for this study.

The study on human tissue was observational and retrospective. It was limited to the number of available samples of breast carcinomas that had been treated with neoadjuvant endocrine therapy and undergone surgical resection at Mayo Clinic and from which slides were available in a tissue bank. Non-treated human tissue samples were obtained at surgery at CORI Cancer Center from a tissue bank.

The samples include lobular, ductal and special subtypes of carcinomas with dissimilar grades of tissue differentiation. Patient characteristics are presented in Table 1 for treated patients and Table 2 for untreated patients. Treated-patients received neoadjuvant therapy with a mean of 150 days (range, 14 to 381) with different endocrine drugs included (Table 1). Clinical staging of patients ranged from stage I to IV. Tumor size was determined clinically with a caliper in some cases, and with imaging (ultrasound and Magnetic Resonance Imaging) in others.

To evaluate the therapeutic response, we compared tumor size before starting neoadjuvant therapy and immediately before surgery. With these data we calculated the percentage of tumor reduction after therapy for each sample. A variety of responses were seen, ranging from an increase of the tumor size in 47% to a reduction of almost 83% of the tumor mass. The samples were arbitrary classified according to a 30% cut off of tumor reduction. A pathologist performed all analysis and quantification of parenchymal and stromal markers in patient samples.

Supplementary material 1

Hormone-dependent (HD) C4-HD and 59-HD tumors were maintained by a subcutaneous depot of MPA (20 mg). The hormone-independent (HI) tumor variants C4-HI and 59–2-HI were derived from C4-HD and 59-HD tumors, respectively, and grown in mice that had not been treated with MPA. 32–2-HI and C7–2-HI are other transplantable HI ductal mammary carcinomas from the MPA murine model. All tumor variants used in this study regress when treated with antiestrogens or antiprogestins.

Tumors were maintained through serial syngeneic subcutaneous transplants by trocar into the right inguinal flank of mice and treated, if necessary, with MPA. Tumor size (length and width) was measured with a Vernier caliper. When tumors reached a size of approximately 50mm2, treatments were started.

MFP (Sigma Aldrich) and TAM (Gador Argentina) were inoculated subcutaneously. WORT (Sigma Aldrich) and RAPA (LC Laboratories) were administrated by intraperitoneal injection. For treatments of 24 h or more, drug administration was done every 24 h and 1 h before sacrifice, except for ICI (AstraZeneca) that was given as a single subcutaneous depot of 5 mg.

Supplementary material 2

IBH-6 and T47D cells were cultured at 37°C in 10% Fetal Calf Serum (FCS)-DMEM/F12 medium.

Cell counting: IBH-6-Acl4 and IBH-6-myrAkt1 cells were seeded on 12-well plates at a density of 4×10^4 cells/well. After attachment, the cells were starved with DMEM-2% stripped FCS for 24 h and then incubated for 6 days with the experimental solutions to be tested. The solutions were replaced every other day. When treatments were finished, the cells were trypsinized, centrifuged, resuspended in 0.5 ml of media, and counted using a hemocytometer.

For T47D xenografts, NOD/SCID female mice were implanted subcutaneously with silastic pellets containing 17β-estradiol (0.25 mg) and injected subcutaneously in the right inguinal flank with 1×10^7 T47D-Acl4 or T47D-myrAkt1 cells mixed with Matrigel (BD Biosciences). One week after cell injection tumor-bearing mice were randomized into the different experimental groups. MFP was administrated as silastic pellets (5 mg) implanted subcutaneously in the back of the animals.

Supplemental material 3

Tumors were fixed in 10% formalin, paraffin-embedded (FFPE) and sectioned into 5 µm for histochemical analysis. The histopathological features of both tumor parenchyma and stroma were evaluated in hematoxylin and eosin (H&E)-stained slides.

Apoptosis and mitosis were counted by direct evaluation of 10 high-power fields (HPF) in at least three independent tumors per experimental group. Both parameters were expressed as percentage of cells in mitosis or apoptosis/total number of cells in each high-power field.

For necrotic and stromal area quantification, H&E stained sections were photographed at a 200X magnification to cover the entire tumor slices, using a Nikon Eclipse E800 Microscope with ACT-2U (for Nikon) software. Subsequently, images were assembled with PanaVue ImageAssembler software, and necrotic and stromal areas were determined in the resulting photographs using ImageJ software. Both parameters were expressed as percentage of necrotic or stromal area/total section area in at least three independent tumors per experimental group.

Supplementary material 4

Primary antiserum was detected after incubation with a biotinylated secondary antibody (Vector Laboratories Inc.) using the Vectastain Elite ABC Kit (Vector Laboratories Inc.) and the diaminobenzidine (DAB) Chromogen and Substrate Buffer (Dako, Agilent Technologies). After IHC, the specimens were counter-stained with hematoxylin, dehydrated, and mounted.

Ki67-positive cells were counted in ten HPF in at least three independent tumor sections per experimental group, and expressed as the percentage of positive events over total cell number per HPF.

For active caspase 3 quantification stained slides were scanned at a 200X magnification (Aperio ScanScope AT (Aperio Technologies)). Stain intensity was determined in at least three independent tumor sections using the Positive Pixel Count algorithm from Image Scope software (Aperio Technologies). Caspase 3 index was expressed as the ratio between staining intensity and total tumor area.

Staining for αSMA and pS6 in human specimens was measured with a four-value intensity score (0, 1+, 2+, and 3+) and the percentage of the reactivity extent (range, 0 to 100). Final scores were obtained by multiplying both intensity and extension values (range, 0 to 300). For pS6, parenchymal and stromal scores were calculated separately.

Supplementary material 5

Frozen tumors were cut using a cryostat obtaining 18 µm thick sections. Sections were air-dried, fixed in 4% paraformaldehyde (PFA) in PBS, permeabilized with 2.5% Triton X-100 in methanol, and blocked by incubation with PBS containing 10% fetal calf serum (blocking buffer). Sections were then incubated with primary antibody dissolved in blocking buffer overnight at 4°C. After washing with PBS, sections were incubated with fluorescein-conjugated secondary antibody for 1 hour at room temperature in blocking buffer. Nuclei were stained with propidium iodide (PI; Sigma Aldrich) and slides were mounted with Vectashield (Vector Laboratories Inc.). Sections were analyzed under a Nikon Eclipse E800 Confocal Microscope.

CD31 stain quantification was performed by counting the number of CD31-positive foci in five photographs at 200X magnification in at least three independent tumors per experimental group.

For lectin staining: tumor-bearing mice were injected with fluorescein labeled Lycopersicon esculentum Lectin (Vector Laboratories Inc.) 10 min before sacrifice. Subsequently, mice were perfused with a cold saline solution (0.9% NaCl) followed by 4% PFA, and tumors were excised and cryopreserved for fluorescence detection.

Supplementary material 6

Tumor primary cultures: After isolation, cells were seeded on top of Matrigel in 10% FCS-DMEM/F12 medium. After 48 h the medium was removed, and cells were exposed to MFP, WORT, MFP+WORT, or dimethyl sulfoxide as vehicle for another 48 h. All treatments were carried out in 2% charcoal stripped FCS-DMEM/F12 medium, and replaced every 24 h. At the end of the treatment cells were fixed with paraformaldehyde PFA 4% for 20 minutes at room temperature. Fixed clusters were permeabilized with 5% triton X-100 in PBS, and incubated with blocking buffer. Primary and secondary antibodies incubations, nuclei staining, mounting and analysis were performed as explained in immunofluorescence for tumors.

For proliferation and apoptotic indexes Ki67 and caspasa 3-stained cell clusters, respectively, were photographed at a 200X magnification (obtaining approximately 5 clusters per photograph), using a Nikon Eclipse E800 Confocal Microscope. Proliferation index was calculated as the number of Ki67-positive cells over

the total number of cells in each cluster. Apoptotic index was calculated as the number of clusters with at least 4 caspase 3-positive cells over the total number of cell clusters. Both proliferation and apoptotic indexes were set arbitrarily set to 1.0 in vehicle-treated groups.

Apoptosis was also studied by staining the cells with Acridine orange and ethidium bromide (AO/EB). Clusters were treated for 10 seconds with AO/EB 4mg/ml in PBS and immediately visualized with a Nikon Eclipse E800 Confocal Microscope. AO/EB staining that intercalates with double-stranded DNA was used to detect nuclear changes and apoptotic body formation. Live cells fluoresce green (with AO) and dead cells fluoresce orange/red (with EB).

Supplementary material 7

For western blots (WB) total protein extracts were prepared by homogenizing tumor samples in RIPA buffer. Protein concentration was measured using the Lowry method. Samples were mixed with 4 volume of sample buffer containing β-mercaptoethanol and boiled for 5 minutes. 100 µg of each sample were then separated in 8%, and 10% sodium dodecyl sulfate-polyacrylamide gel electrophoresis mini gels (Bio-Rad) and transferred to nitrocellulose membranes (Hybond-ECL; Amersham Biosciences). The membranes were blocked for 2 h in 5% bovine serum albumin (BSA) in tris-buffered saline (TBS) at room temperature. Primary antibodies were diluted in blocking buffer TBS containing 0.1% Tween-20 (PBST) and 2.5% BSA and were incubated overnight at 4°C. Band intensity was quantified using the Image J software. Relative densitometric units of phosphorylated protein's bands were normalized using total proteins, with the density of the control group bands set arbitrarily set to 1.0.

Primary antibodies

Phosphorylated Ser473 Akt antibody (33437), total Akt (8312) and PI3K (602) were purchased from Santa Cruz Biotechnology; phosphorylated Ser240/244 S6 (2215), Ser235/236 S6 (2211) and E-Cadherin (3195) from Cell Signalling Technology, Ki67 (15580); αSMA (5694), active caspase 3 (2302), integrin a6 (19765), collagen I (6308) and MUC-1 (45167) from Abcam; CD31 antibody (550274) from BD Pharmigen; laminin-1 from Ly Laboratories. For patient samples studies aSMA (M0851), CD31 (IS61030–2) and Ki67 (M7240) from Dako, and active caspase 3 (9664) from Cell Signaling Technology, were used. Both phosphorylated Ser240/244 S6 (2215) and Ser235/236 S6 (2211) labelled parenchymal and stromal protein both in mouse and human tissues.

Author contributions

M.L.P., M.R., C.L. and V.N. designed experiments. M.L.P. and M.R. performed experiments. M.L.P. assisted with manuscript writing. M.M. stained and analyzed human samples. M.S.M. collected and stained human samples. M.J.R and M.C.P assisted with experiments. D.L.K, M.H.F., M.P.G. and J.C.B. provided human samples and data; C.L. contributed with discussion. D.C.R. contributed with discussion and manuscript writing. V.N. settled the research and wrote the manuscript.

SUPPLEMENTARY FIGURES

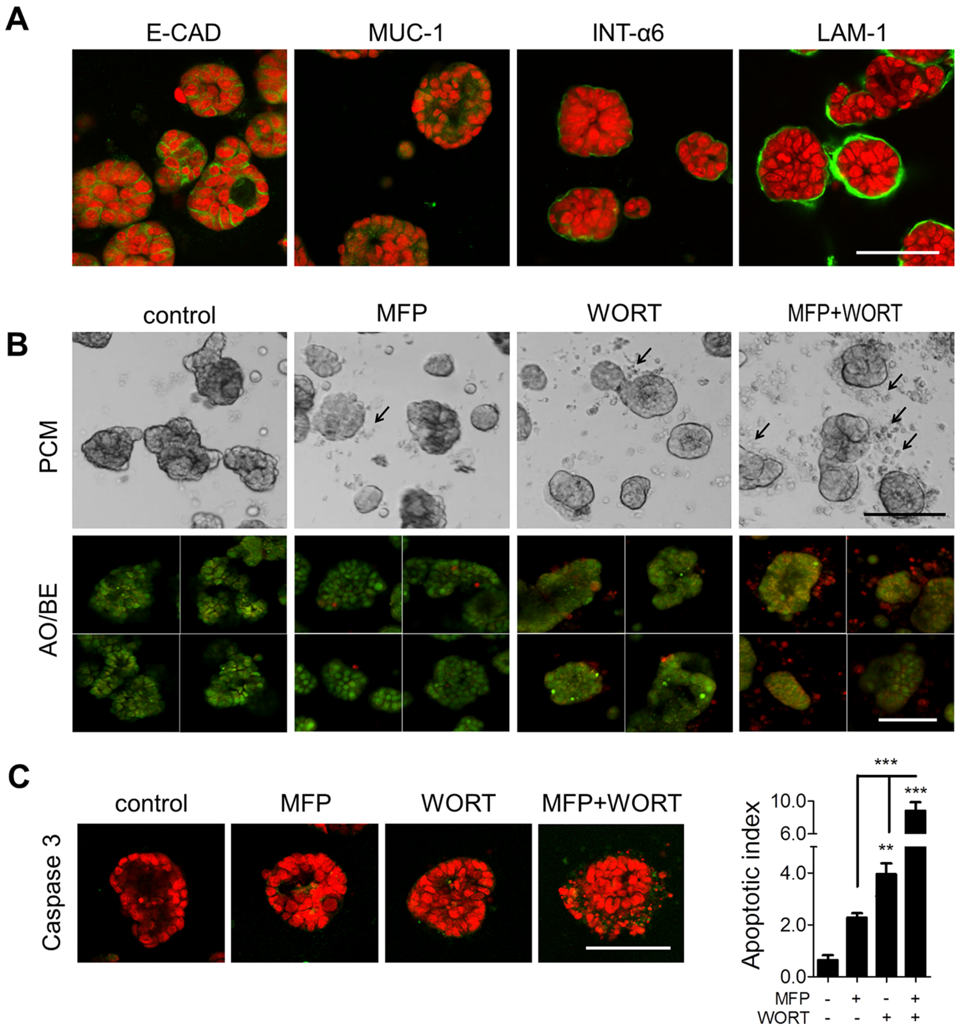

Supplementary Figure S1: WORT enhancement of MFP-induced apoptosis is preserved on isolated C4-HI tumor cells growing on 3D Matrigel. Primary epithelial cells isolated from C4-HI tumors were placed on top of Matrigel and cultured for 48 h. The cells developed organized and polarized clusters of about 100 μm size. **A.** Confocal images of IF for polarity markers show appropriate lateral localization of E-cadherin (E-CAD), apical localization of mucin-1 (MUC-1), continuous basal localization of integrin-α6 (INT-α6) and laminin-1 (LAM-1). **B.** Phase contrast microscopy of C4-HI clusters treated for 48 h with MFP (0.01 μM), WORT (1 μM), the combination or vehicle (control). In combined-treated clusters cell detachment was increased (arrows). Cell death was confirmed by Acridine Orange/Ethidium Bromide (AO/EB) differential staining. **C.** Left: Confocal images for caspase 3 immunostaining confirmed that increased cell death after combination therapy is through apoptosis. Right: Apoptotic index determined in cell clusters. Data are representative of 3 independent experiments. Bar: 100 μm.

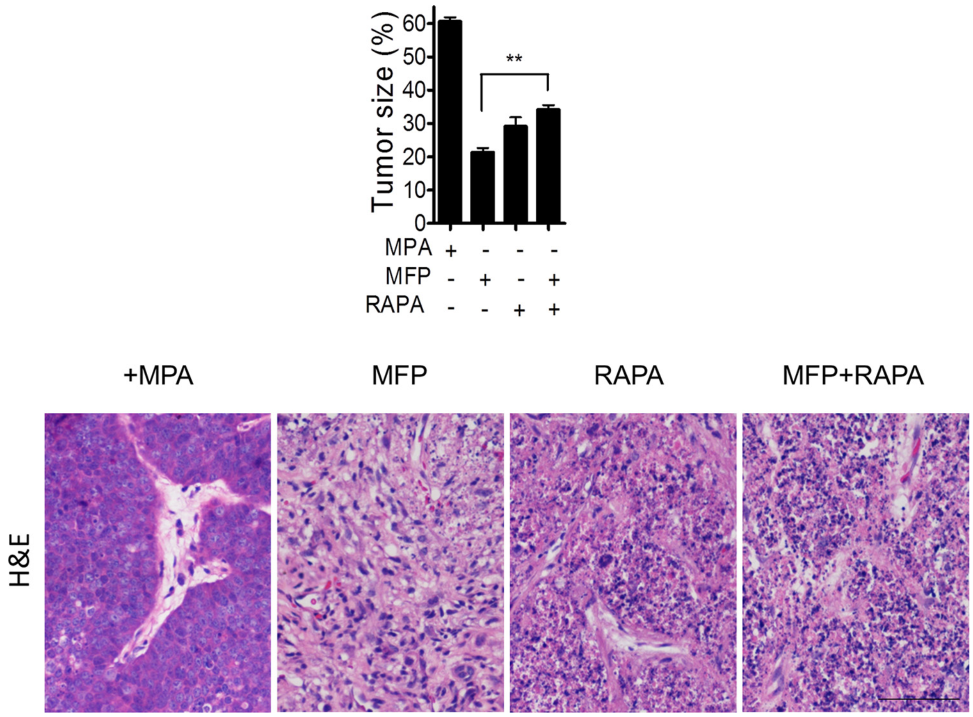

Supplementary Figure S2: RAPA treatment interferes with the stromal reaction induced by endocrine therapy in C4-HD tumors. Mice carrying C4-HD tumors as described in Figure 2 were treated with MFP, RAPA (5mg/kg/day) or the combination. **Top:** Final tumor size of each experimental group after 48 h of treatment. MFP-treated tumors showed smaller size compared to WORT or MFP+RAPA-treated ones. Bottom: H&E staining in FFPE tumor sections revealed greater tissue remodeling in MFP-treated tumors. Bar: 100 µm.

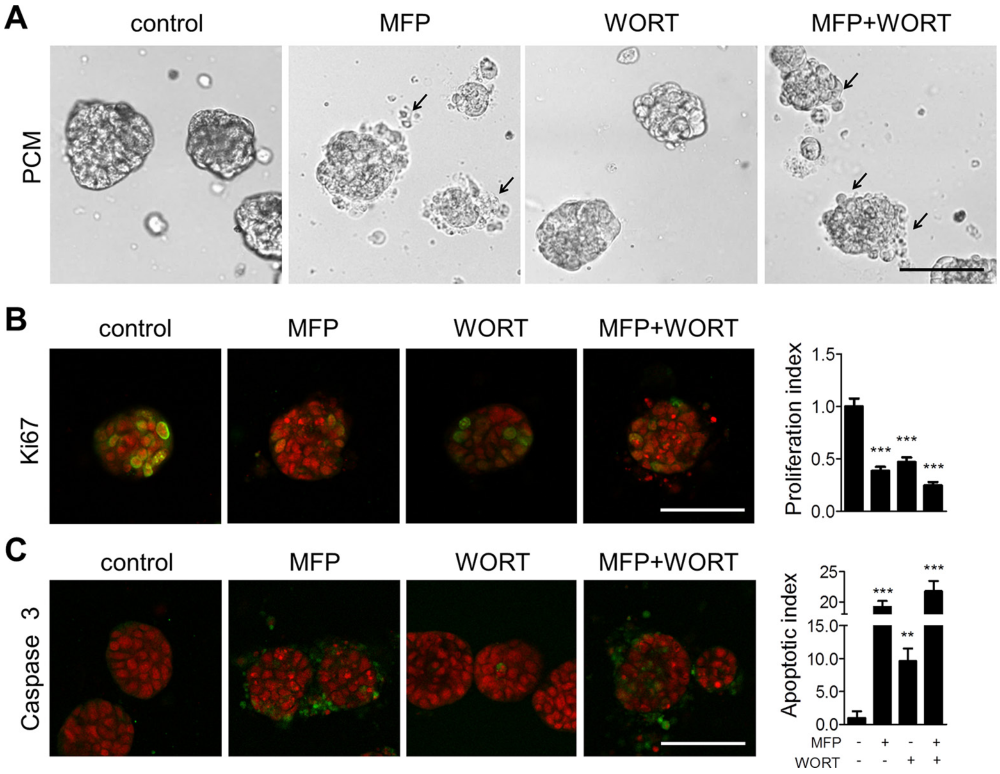

Supplementary Figure S3: MFP-induced apoptosis of isolated C4-HD tumor cells is not improved by the combination with WORT. Primary epithelial cells isolated from C4-HD tumors were placed on Matrigel and cultured for 48 h with MFP (0.01 µM), WORT (1 µM), the combination or vehicle (control). **A.** Phase contrast microscopy shows organized cell clusters of about 100 µm size and increased cell detachment (arrows) after MFP or MFP+WORT treatments. **B.** Confocal images for Ki67 immunostaining and Ki67 index show that MFP, WORT or the combination induced cytostasis of tumor cells in a similar grade. **C.** Caspase 3 staining confirms that detached cells from MFP- and MFP+WORT-treated clusters were apoptotic. Caspasa 3 index shows that MFP and MFP+WORT increased apoptosis in a similar grade. Data are representative of 3 independent experiments. Bar: 100 µm.
